# Temporal escape–adaptation to eutrophication by *Skeletonema marinoi*

**DOI:** 10.1093/femsle/fnac011

**Published:** 2022-02-08

**Authors:** Malin Olofsson, Anna-Karin Almén, Kim Jaatinen, Matias Scheinin

**Affiliations:** Department of Aquatic Sciences and Assessment, Swedish University of Agricultural Sciences, Box 7050, 750 07 Uppsala, Sweden; Tvärminne Zoological Station, University of Helsinki, J.A. Palménintie 260, 10900 Hanko, Finland; Nature and Game Management Trust Finland, Degerbyvägen 176, 10160 Degerby, Finland; Tvärminne Zoological Station, University of Helsinki, J.A. Palménintie 260, 10900 Hanko, Finland; Department of Environmental Protection, City of Hanko, Santalantie 2, 10900 Hanko, Finland; Pro Litore, Långgatan 13, 10620 Ekenäs, Finland

**Keywords:** Baltic Sea, resting stages, elevated temperatures, climate change, diatoms, eutrophication

## Abstract

Diatoms commonly set off the spring-bloom in temperate coastal environments. However, their temporal offset may change in regions subject to nutrient enrichment, and by peaking earlier, such populations can maintain their position in the vernal plankton succession. We tested whether the marine keystone diatom *Skeletonema marinoi* can accomplish this through thermal evolutionary adaptation. Eight geographically separated subpopulations, representing hydromorphologically and climatologically similar inlets displaying a range of trophic states, were compared in a common-garden experiment. At early-spring temperatures, both doubling times and variation coefficients thereof, correlated negatively with the trophic state of the environment of origin, indicating selection for fast growth due to eutrophication. At mid-spring temperatures, the relationships were reversed, indicating selection in the opposite direction. At late-spring temperatures, no significant relationships were detected, suggesting relaxed selection. Subsequent field observations reflected these findings, where blooming temperatures decreased with trophic state. Natural selection thus moves along with eutrophication towards colder temperatures earlier in the spring, favouring genotypes with the capacity to grow fast. The thermal niche shift demonstrated herein may be an evolutionary mechanism essentially leading to trophic changes in the local ecosystem.

## Teaser

The study demonstrates the potential of a key diatom species to adapt to eutrophication by shifting its thermal niche, using a combination of field and laboratory observations.

## Introduction

Anthropogenic impacts including eutrophication affect all ecosystems on Earth (Steffen *et al*. 2015). Our current understanding of the impacts of human activities on the natural world indicates that many organisms struggle to adapt to the rapid changes that humans impose (Parmesan 2006). Species unable to adapt, are forced to move their distribution ranges, or they perish (Chen *et al*. 2011). While findings of evolutionary responses to global change are scarce in vertebrates (e.g. Charmantier and Gienapp 2014), species with shorter generation times may be more able to exhibit evolutionary responses to environmental change (Hoffmann and Sgró 2011). Chain-forming diatoms are key organisms in marine environments by being responsible for half of their oxygen production, acting as ballast (in the marine carbon pump), and being food for grazers (Nelson *et al*. 1995). With generation times of 1–2 days during exponential growth (Olofsson *et al*. 2019a), days to weeks in the field (Olofsson *et al*. 2019b, Bergkvist *et al*. 2018), and with large population sizes, they may adapt to changing conditions at a rapid pace. Diatom populations may therefore act as indicators of anthropogenic impacts exerting selective pressures that may shape aquatic food webs under climate change.

Many key species of diatoms form spring-blooms (Nelson *et al*. 1995, Bergkvist *et al*. 2018), despite the physiological limitations that colder temperatures and fewer daylight hours pose on replication rates. The main advantage provided by this taxonomically widespread (and thus primeval) trade-off strategy is that spring-blooming diatoms may avoid a complex set of different pressures such as the strong competition for resources, the higher grazing pressure, and abundant diseases common during later stages of the seasonal succession (Smayda 1997). Before a population peaks, its replication rate (driven primarily by temperature, light, and nutrients) exceeds that of total losses. After the peak, this relationship is inverse, meaning a negative rate of population change (Reynolds 2006, Thackeray *et al*. 2008). When surrounding conditions are less favourable, many species of diatoms form resting stages in the bottom sediment. These resting stages are known to benefit the population by increasing its genetic diversity (Sundqvist *et al*. 2018), and they reflect the population during the senescent bloom. Newly hatched resting stages of diatoms can therefore be efficiently used in evolutionary laboratory studies addressing adaptation (Härnström *et al*. 2011, Olofsson *et al*. 2019a).

**Figure 1.**
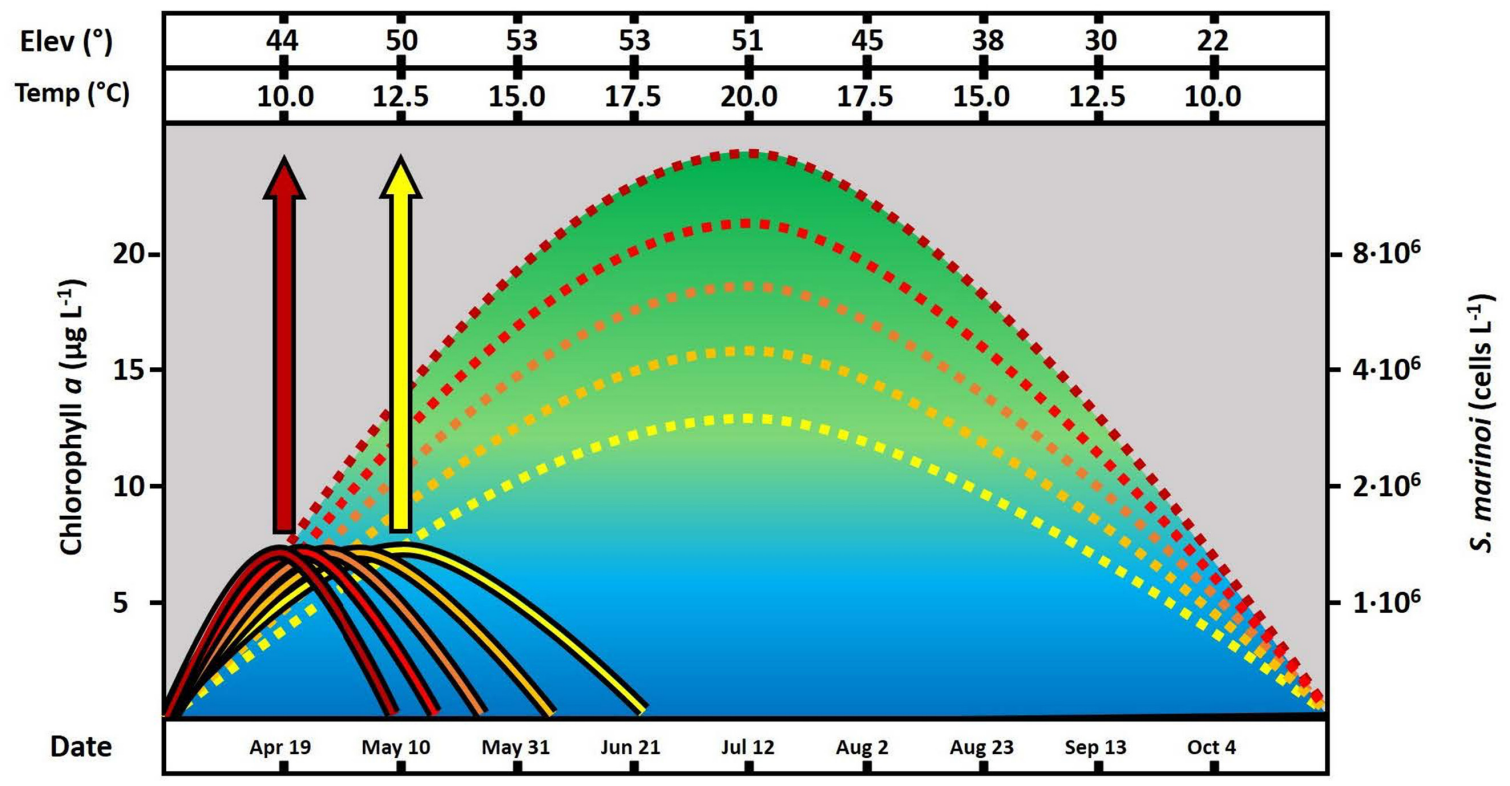
A schematic view of the seasonal dynamics of Chl *a* (dashed) and spring-blooming *Skeletonema marinoi* (solid) in coastal environments representing different trophic states (colour gradient from yellow to dark red). The arrows indicate the time at which selection pressure for rapid growth is at its highest in mesotrophic (yellow) vs. eutrophic (dark red) conditions. Elev = solar elevation angle at noon and Temp = temperature.

The Baltic Sea is a semi-enclosed brackish-water basin stressed especially by coastal eutrophication (Reusch *et al*. 2018). Since the eutrophication status of the coastal ecosystem displays considerable spatial variability, different phases of this progression can be observed simultaneously. This study took place in shallow semi-isolated inlets in southwestern Finland (North East Baltic Proper). These physically distinctive systems with clear-cut boundaries represent varying degrees of eutrophication, i.e. trophic states (Munsterhjelm 2005, Scheinin *et al*. 2013). In shallow waters, phytoplankton biomass varies seasonally like a downward parabola, its width driven by temperature and light, and height by trophic state (Fig. 1; Harding *et al*. 2019). Here, the peak phytoplankton biomass reflects the results of environmental selection from e.g. grazing, temperature, and light, which is equal to the species' realized niche (Hutchinson 1957). In contrast, examining the growth rates of a species under laboratory conditions would reflect the fundamental niche, i.e. its maximum capacity.

We tested if the keystone diatom *Skeletonema marinoi* has adapted to coastal eutrophication through a thermal niche shift—to maintain its position in the vernal succession by (i) reviving resting stages from shallow inlets representing different degrees of eutrophication, and examining their potential to grow in temperatures representing early, intermediate and late spring, by (ii) addressing the extent of selection within each of the temperatures, and by (iii) determining *in situ* population density-peak temperatures of the diatom in the shallow inlets.

## Material and methods

### Site description and field sampling

**Figure 2.**
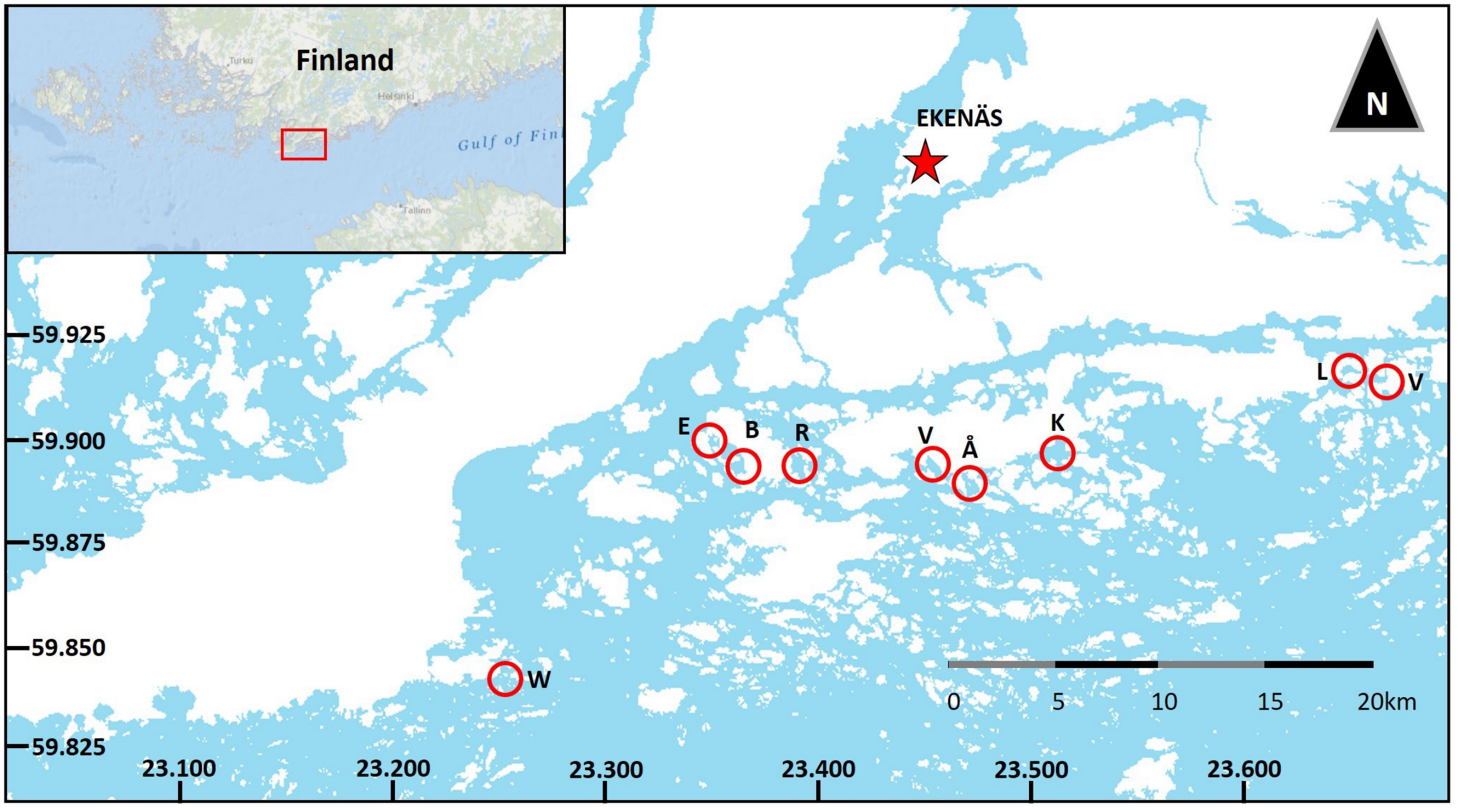
Map of the shallow inlets used in the experiment. From left to right; Ekholmsfladan, Björkholmsfladan, Ramsängsfladan, Hälsingfladan, Åkernäsfladan, Kopparöfladan, Lillfladan, and Västerfladan. Water for the experiment was collected from a monitoring site (W).

To summarize the procedure, eight shallow inlets (ca. 1 m depth) located in the North East part of the Baltic Proper were sampled (Fig. 2) during 2016 (laboratory experiment) and 2017 (field confirmation). All inlets are classified as juvenile flads with essentially similar morphology (Munsterhjelm 2005). This also means that the inlets are equally and only slightly isolated from the surrounding waters. As the inlets are also located within the same archipelago zone and region (Bonsdorff *et al*. 1997), they can be expected to be similar also in terms of their temperature and salinity dynamics. The water (5 PSU) used for the experiment in 2016 was collected nearby Tvärminne zoological station (59°51′20″N, 23°15′42″E), monitoring site W (Fig. 2). In order to avoid clogging, the filtration was conducted sequentially through 100, 50, 5 (Parker domnick hunter, Texflow Filter Cartridges, VWR International, Helsinki, Finland), and 0.2 µm filters (Sartobran 300, VWR International, Helsinki, Finland). The sterile filtrate was thereafter used to prepare f/2 media (Guillard 1975; with added silicate) for hatching and culturing the clones.

### Sediment samples and hatching of resting stages

Sediment samples containing resting stages of *S. marinoi* were collected in June 2016 from the previously mentioned eight shallow inlets. Five cm deep samples, corresponding to about 3–5 years of sedimentation; Heiskanen and Leppänen 1995), were collected after the spring bloom to avoid vegetative cells and seasonal variation in life stages. Fifteen to twenty such core samples (40 mL) were collected around the centre of each inlet, within a circle with a 50 m +/– 10 m radius and from a depth of 2.0 m +/– 0.2 m. In order to avoid any water intrusion, a custom-made core sampler was placed 1–2 mm below the sediment surface in such a way that the edges of the cylinder and the head of the piston were in line with each other. While the cylinder was lowered into the sediment, the piston was constantly kept in its place. After reaching the desired depth, the sampler was lifted to the water surface with its mouth facing directly downwards. The sediment samples were stored dark at 4°C until hatching.

The germination of *S. marinoi* resting stages was performed by suspending 1 g sediment into 12 ml f/2 media in transparent (15 ml) Falcon vials. After letting the sediment settle for 2 h, 1 ml of suspension from each vial was transferred into 24-well plates. The growth plates were kept at 5°C in a 12:12 h light:dark cycle and 80 µmol photons m^–2^ sec^–1^. When diatom chains appeared after a few days, a single chain per well was isolated using a microscope (Leica DM IL, Buffalo Grove, IL, USA) and a 20 µl pipette (Finnpipette, VWR International, Helsinki, Finland). The target species were identified by light microscopy, however, with a hypothetical risk of also including *S. subsalsum* which has occasionally been found in the Gulf of Finland but in considerably lower salinities (Hällfors 2004).

Ten isolated chains from each bay were transferred into new 24-well plates, one per well, and kept at 15°C in the same light to dark cycle. When each well was full of chains, five clonal lineages from each of the eight locations were randomly selected and transferred into separate 50 ml sterile culture flasks (Greiner Bio-One T-25, VWR International, Helsinki, Finland). The clonal lineages were thereafter re-inoculated regularly to keep them in the exponential growth phase until the growth experiments following Scheinin *et al*. (2015). Although every isolate started growing successfully, a total of six monoclonal lineages were discarded prior and during the experimental treatments due to suspected contaminations. The discarded lineages represented variation in the trophic state of their ancestral environment in a random fashion, as they were from different locations.

### Field measurements

In order to determine the eutrophication status of the shallow inlets, chlorophyll (Chl) *a* and inorganic nutrient samples were collected weekly from mid-June until mid-August during 2016. The inorganic nutrient concentrations were positively and strongly correlated with those of Chl *a* (Tables 1 and S1). Accordingly, this conventional indicator for the trophic status of the environment (Nixon 1995) can be considered a valid proxy for defining the degree of eutrophication (Carlson and Simpson 1996) in the studied inlets. Measuring environmental parameters during summer directly reflects the situation during spring as demonstrated by the unimodal seasonal dynamics (Harding *et al*. 2019). Water (50 ml) for chlorophyll analysis was filtered onto GF/F (Whatman, VWR International, Helsinki, Finland) filters and frozen in scintillation vials. The samples were dissolved with ethanol (96.1%) and Chl *ɑ* was determined by fluorometry (Varian Cary Eclipse spectrofluorometer, Varian Inc., Mulgrave, Victoria, Australia) with a plate reader. Phosphate was determined according to Grasshoff *et al*. (1999), nitrite and nitrate according to (Schnetger and Lehners 2014), using vanadium(III)chloride as reduction agent for nitrate, and silicate according to Koistinen *et al*. (2018), and analysed using an automatic analyser (Aquakem 250, Thermo Fisher Scientific Oy, Vantaa, Finland). Ammonium was determined by manual spectrometric method and measured with a Hitachi U-1100 spectrophotometer (Hitachi, Ltd. Tokyo, Japan). The procedure followed ISO 7150/1–1984 method that is based on the formation of indophenol by sodium salicylate and hypochlorite in the presence of sodium nitroprusside. The correlation analyses incorporated all the measured inorganic nutrients and Chl *ɑ* provided in µg per liter, oxygen in mg per liter and saturation in %, and turbidity in nephelometric turbidity units (NTU).

**Table 1.** Pearson linear correlation coefficients for environmental variables. Significance level was set to ɑ > 0.05, which is shown in boldface, *n* = 101. Data used in the correlation can be found in Table S1.

Variables	Ammonium	Nitrite + nitrite	Phosphate	Silicate	Temperature	Salinity	Oxygen conc.	Oxygen sat.	Turbidity	pH	Chl a
Ammonium	**1**	0.554	0.671	0.695	–0.668	0.274	–**0.832**	–**0.779**	**0.739**	–**0.822**	0.480
Nitrate + nitrite	0.554	**1**	**0.922**	0.591	–0.006	–0.201	–**0.751**	–**0.822**	**0.729**	–0.521	**0.855**
Phosphate	0.671	**0.922**	**1**	**0.719**	–0.120	–0.048	–**0.866**	–**0.921**	**0.889**	–0.601	**0.915**
Silicate	0.695	0.591	**0.719**	**1**	–0.237	0.041	–**0.713**	–**0.739**	**0.887**	–0.693	0.703
Temperature	–0.668	–0.006	–0.120	–0.237	**1**	–0.639	0.485	0.325	–0.251	0.547	0.061
Salinity	0.274	–0.201	–0.048	0.041	–0.639	**1**	–0.018	0.094	–0.002	0.145	–0.020
Oxygen sat.	–**0.832**	–**0.751**	–**0.866**	–**0.713**	0.485	–0.018	**1**	**0.984**	–**0.909**	**0.882**	–**0.728**
Oxygen conc.	–**0.779**	–**0.822**	–**0.921**	–**0.739**	0.325	0.094	**0.984**	**1**	–**0.939**	**0.841**	–**0.810**
Turbidity	**0.739**	**0.729**	**0.889**	**0.887**	–0.251	–0.002	–**0.909**	–**0.939**	**1**	–**0.767**	**0.864**
pH	–**0.822**	–0.521	–0.601	–0.693	0.547	0.145	**0.882**	**0.841**	–**0.767**	**1**	–0.421
Chl a	0.480	**0.855**	**0.915**	0.703	0.061	–0.020	–**0.728**	–**0.810**	**0.864**	–0.421	**1**

### Experimental procedure

Prior to each experimental treatment, the isolated strains of *S. marinoi* were acclimated to the new conditions for two full batch cycles (or about 12 generations) per any 2.5°C change in temperature, kept under exponential growth, whereafter they were transferred to a final concentration of 100 cells ml^–1^ (Scheinin *et al*. 2015). Five flasks for each treatment and from each shallow inlet were incubated in water baths adjusted to 10°C, 12.5°C, and 15°C. These temperatures were chosen to represent early, mid-, and late spring, i.e. different phases when *S. marinoi* is present in this environment (Fig. 1). The flasks were inverted every 4 h for homogenisation and their positions in the water bath rotated to experience similar light conditions. Growth-curves were performed prior to the experiment to ensure harvest would occur within the exponential growth phase for all temperatures. After 72 h, cell density was microscopically (Leica DM IL) determined (Utermöhl 1958) using 200-fold total magnification, while growth rates/doubling times were calculated as ln2/(ln (d2/d1))/t (Scheinin *et al*. 2015).

### Blooming temperature in the field

In 2017, biweekly field sampling was performed between mid-April and late June (10 am–2 pm), starting one week after ice breakup, from the same shallow inlets as in 2016. Water was collected with a 2-L Limnos sampler (Limnos Oy, Turku, Finland) from 1 m depth close to the midpoint (depth ca 2.0 m) of each bay. Water temperature was measured by using a portable sonde (proODO Optical Dissolved Oxygen Instrument, YSI Incorporation, Yellow Springs, OH, USA), and Chl *a* samples were collected as described above. Non-filtered phytoplankton samples (50 mL) were preserved with acid Lugol's solution prior to analyzing them microscopically (as described above) for *S. marinoi*. Each sample was analyzed within three months after its collection in a sequence of 2 mL subsamples until reaching a total of 30 chains per sample or a total volume of 10 mL. The biovolume (μm^3^ L^–1^) of the target species was estimated based on Olenina *et al*. (2006).

### Data handling and statistical analyses

We set off to answer the three questions addressed by, first, constructing a linear mixed-effects model where doubling time was explained by treatment temperature (in the laboratory) and average Chl *a* concentration (in the field) and the two-way interaction between these two variables. Strain identity was included in the model as a random effect to correct for potential pseudoreplication arising from repeated observations from the same strains.

**Figure 3.**
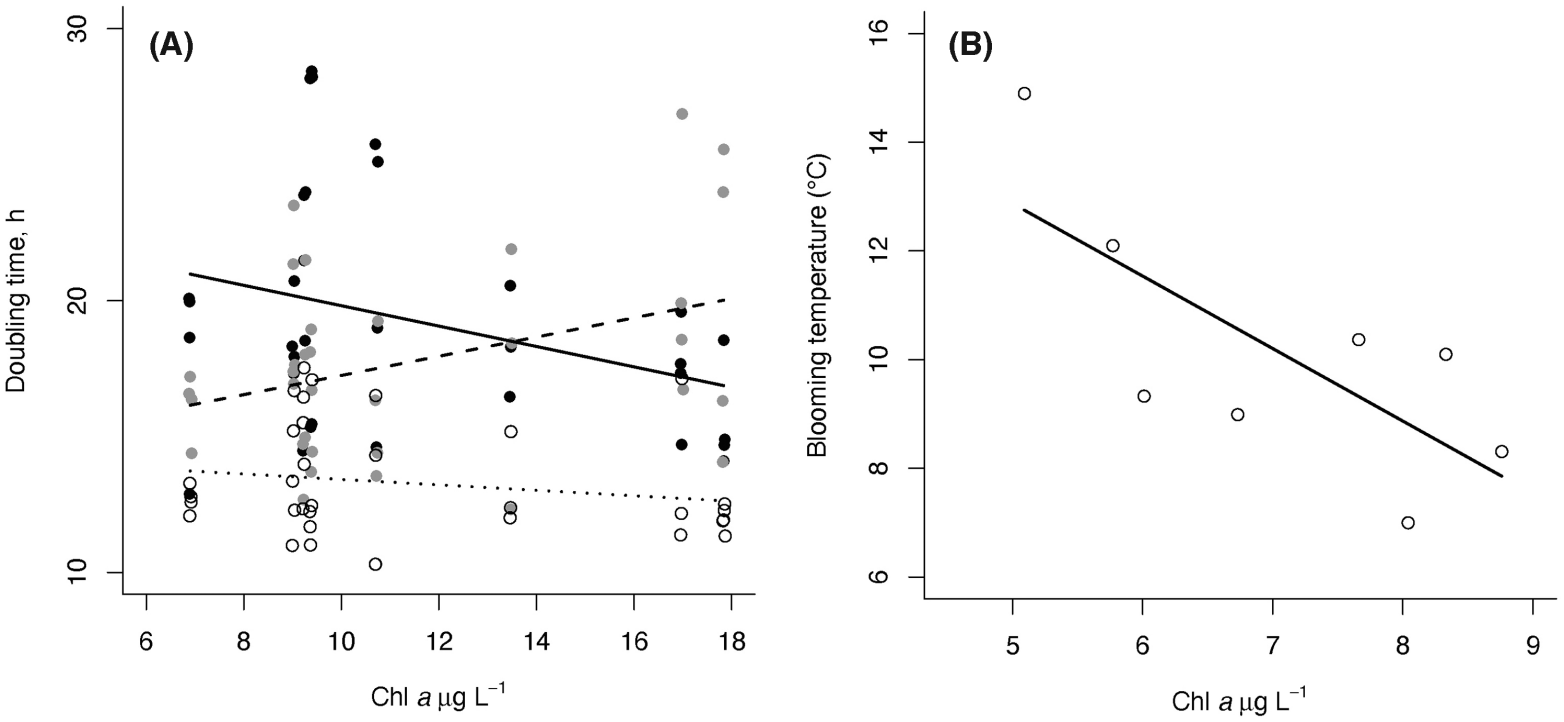
**(A)** The doubling rates of *Skeletonema marinoi* in the three different treatments (10°C: black line and black filled circles, 12.5°C: dashed line, grey-filled circles, 15°C: dotted line and open circles) and **(B)** the *in situ* blooming temperature of eight *S. marinoi* populations, based on biovolume (Table S3), both related to the degree of eutrophication (using Chl a as a proxy) of their natal bay.

Second, to test the presence of selection for a given temperature-trophic state combination in the sampled population we examined the effect of eutrophication and temperature treatment on the coefficients of variation for doubling time exhibited by subsamples of *Skeletonema* sampled from each shallow bay present in the study. Based on the first model we found that the experimental treatment temperatures 10°C and 12.5°C yielded opposite responses to the level of eutrophication over the gradient studied, whereas the 15°C treatment did not exhibit any detectable response to eutrophication in our experiment (Fig. 3A and Table S2). Because of this, we constructed a linear model in which the population specific doubling time was explained by temperature treatment and Chl *a* concentration. In this analysis we only included the temperature treatments yielding a response in the experiment, i.e. those of 10°C and 12.5°C.

Thirdly, we tested whether the blooming temperature of *S. marinoi*, defined as the biovolume-weighted mean temperature (*sensu* Hampton *et al*. 2014), was related to the trophic state of the environment. This was done by constructing a linear model where the blooming temperature in each inlet was explained by its average Chl *a* concentration during the springtime period when *S. marinoi* could be detected in the water column in any of the inlets. The residuals of all analyses adhered to the assumption of normality and all analyses were conducted in the software R 3.6.0 (R Core Team 2019).

## Results and discussion

The chain-forming diatom *S. marinoi* often dominates spring blooms in temperate waters (Godhe *et al*. 2016, Bergkvist *et al*. 2018). We hypothesized that populations of this species can move their thermal niche as a result of eutrophication. In concert, we detected a significant interaction between treatment temperature and the trophic state of the environment of origin on the growth rate of *S. marinoi* populations (Chi2 = 11.14, *P* = 0.004; Fig. 3A and Table S2). This interaction shows that the 10°C (b = –0.08, t = –2.40, *P* = 0.02) and 12.5°C (b = 0.07, t = 2.27, *P* = 0.03) temperature treatments elicit opposite effects of nutrient enrichment in different subpopulations of *S. marinoi* (Fig. 3A), suggesting the presence of local adaptation to eutrophication and altered nutrient competition regimes. We did however not detect a response in doubling time to the degree of eutrophication in the 15°C temperature treatment (b = –0.02, t = –0.65, *P* = 0.52).

According to our hypothesis, increased eutrophication leads to an adaptation in *S. marinoi* to proliferate in colder temperatures, as it allows the avoidance of negative effects later in the season. This notion was supported by the negative correlation between doubling time and the trophic state of the environment of origin in the colder, 10°C treatment (Fig. 3A). Supporting the same notion, the corresponding correlation was positive in the warmer, 12.5°C treatment. Growth under laboratory conditions here reflects the fundamental niches of the strains (Hutchinson 1957). In contrast, field observations will in addition to high growth rates reflect niche selection including environmental factors, e.g. grazing, and instead reflect the realized niche (as demonstrated in Fig. 1). Our field data on the inlet-specific blooming temperatures of *S. marinoi* corroborates our experimental findings by showing that the blooming temperatures correlated negatively with the trophic state of the environment, i.e. Chl a concentration (b = –1.33, t = –2.63, df = 6, *P* = 0.04; Fig. 3B and Table S3). The spring-time average Chl a concentration explained 54% of the variation in blooming temperature among the studied shallow inlets. These coherent results suggest a temporal adaptation to eutrophication, which was revealed by using a combination of a realized (in the field) and fundamental (in the laboratory) niche approach.

Natural selection erodes variation in populations, wherefore *S. marinoi* populations under selection should show the least variation in doubling time at the temperature—trophic state combination best representing the natural circumstances found in their bay of origin. Our analysis showed that the strain-specific variation in doubling time was explained by an interaction between temperature and eutrophication (F_2,12 _= 6.79, *P* = 0.01). Variation in doubling time at 10°C was lower with higher trophic state (i.e. higher selection pressure for this trait in their natural environment). This suggests that fast growth in low temperatures can be an advantage in a nutrient rich environment and may thus be favoured by natural selection. In contrast, an opposite response pattern was detected at 12.5°C. From a selective point of view it thus seems important to be able to grow fast in relatively warmer water originating from a nutrient poor environment, which can be an adaptation to grow later in the season when temperatures are higher, compensating for low nutrient availability. This relationship between treatments indicates a threshold in adaptation to different temperature niches and would give the strains from different inlets a thermal niche separation. Thereby, phenotypes that can grow fast in cold water would be favoured by natural selection in relatively nutrient rich water as compared to the nutrient poor, whereas phenotypes that can grow fast in warm waters would be favoured in relatively nutrient poor waters as compared to nutrient rich.

The overall highest growth rates were observed at 15°C, independent of the natural nutrient conditions (Fig. 3A and Table S2). Even though elevated temperatures do increase the metabolic rate of *Skeletonema* under nutrient replete conditions (Marañón *et al*. 2018), it has usually already decreased in abundance when the temperature reaches 15°C (Niemi and Åström 1987). Further, there was an overall high variance in doubling times, and thereby, the natural selection does not seem to have an effect on growth rates here, as they are not associated with any evolutionary advantages or disadvantages. Supposedly, competition between strains is decided by the highest possible temperature before the rest of the environment is non-favourable for *S. marinoi*. However, chain-forming diatoms can be important in nutrient cycling also in late summer, where high growth rates can sustain a less dense population (Olofsson *et al*. 2019b).

Evolutionary change may occur even if its phenotypic consequences are masked by concurrent environmental change. Such cryptic evolution (Cooke *et al*. 1990) can be expected to take place in ecosystems undergoing the complex phenomenon of eutrophication. Considering the phenological responses of diatom populations to eutrophication, their deeply imprinted evolutionary strategy as spring-bloomers and the number of different selection pressures associated with eutrophication, a thermal niche shift can be seen as a highly feasible evolutionary outcome. Although potentially masked by environmental changes, using a dual approach as herein might reveal these changes.

Climate change acts synergistically with eutrophication and future projection for the Baltic Sea includes an earlier onset of the spring bloom due to elevated temperatures (Sommer *et al*. 2012, Kahru *et al*. 2016). Accordingly, elevated seawater temperatures in spring have advanced the timing of the spring bloom in the study region (Wasmund *et al*. 2019), associated with a shorter lag between phyto- and zooplankton biomass peaks (Almén and Tamelander 2020). Although primary production generally increases with eutrophication, the negative effects later in the season override the positive feedback for the spring blooming species. Elevated temperatures and eutrophication can both enhance adaptation to grow earlier in the season, where optimum is to have a perfect timing in terms of light availability, nutrients, and temperature, while negative effects are as low as possible. We demonstrate that *S. marinoi* has the potential to adapt to eutrophication by increasing its growth rate at lower temperatures and thus blooming earlier. This adaptation might have future implications for grazers, and thus, for the food web structure of this coastal ecosystem.

## Supplementary Material

fnac011_Supplemental_File
