# Supplementary material for: Temporal escape–adaptation to eutrophication by Skeletonema marinoi
Source: FEMS Microbiol Lett. 2022 Feb 8;369(1):fnac011. doi: 10.1093/femsle/fnac011 (PMC8973911; doi:10.1093/femsle/fnac011)
Supplement: fnac011_Supplemental_File [file fnac011_supplemental_file.pdf]

Malin Olofsson[1,*], Anna-Karin Almén[2], Kim Jaatinen[3], and Matias Scheinin[2,4,5]

[1]Swedish University of Agricultural Sciences, Department of Aquatic Sciences and Assessment, Uppsala, Sweden
[2]Tvärminne Zoological Station, University of Helsinki, J.A. Palménintie 260, FI-10900 Hanko, Finland
[3]Nature and Game Management Trust Finland, Degerbyvägen 176, FI-10160, Degerby, Finland
[4]Department of Environmental Protection, Hanko, Finland
[5]Pro Litore Association, Raseborg, Finland

*Corresponding author

Table S1 - Field 2016

Site	Site name
Ammo	Ammonium (µg per L)
Nitr	Nitrate (µg per L)
Phos	Phosphate (µg per L)
Sili	Silicate (µg per L)
Temp	Temperature (°C)
Sali	Salinity
OxyP	Average oxygen saturation percent in the field (%)
OxyC	Average oxygen concentration in the field (µg per L)
Turb	Average turbidity in the field
pH-v	Average pH value in the field
Chlo	Average Chl a concentration in the field (µg per L)

Table S2 - Experiment 2016 - in the laboratory

FiDe	Final density *S. marinoi* (cells per ml)
DoTi	Doubling time (h)
Chlo	Average Chlorophyll a concentration in the field (µg per L)
LaTe	Treatment temperature in the laboratory
Site	Site name abbreviations
ClID	Clone-specific identity number

Table S3 - Field 2017

Site	Site name
Week	Sampling week (2017)
TempW	Temperature (°C)
ChlaW	Chlorophyll a concentration (µg per L)
aSKE	*S. marinoi* biovolume (μm^3 per L)

Table S1. Field samples 2016. Site name, ammonium, nitrate, phosphate, and silicate concentration (ug L^{-1}), temperature (°C), salinity, average saturation oxygen concentration (%), average oxygen concentration (ug L^{-1}), turbidity, pH value, and Chlorophyll a (ug L^{-1}).

Site	Ammo	Nitr	Phos	Sili	Temp	Sali	OxyP	OxyC	Turb	pH-v	Chlo
VÄS	5.1	7.3	10.4	227.5	20.5	4.6	98.9	8.7	8.1	7.8	17.8
LIL	3.7	4.4	8.9	276.7	20.7	4.9	107.2	9.5	8.3	8.1	17.0
KOP	2.9	4.8	7.8	149.9	21.1	4.9	120.1	10.5	4.0	8.7	13.5
EKH	4.0	4.1	7.5	178.7	20.1	4.9	110.2	9.9	4.8	8.1	9.0
BJÖ	2.5	3.4	5.2	173.4	20.7	4.8	121.6	10.8	3.4	8.4	9.3
RAM	2.6	2.2	5.0	112.9	21.1	4.5	121.2	10.7	3.2	8.3	6.9
ÅKE	2.3	2.5	5.5	65.5	20.3	5.1	118.0	10.6	3.0	8.5	9.4
HÄL	5.6	3.4	7.0	219.3	19.7	5.2	109.7	9.9	5.9	8.0	10.7

Table S2 - From the laboratory experiment in 2016. FiDe: Final density *S. marinoi* (cells per ml); DoTi: Doubling time (h); Chlo: Average Chlorophyll a concentration in the field (ug per L); LaTe Treatment temperature in the laboratory; Site; ClID: Clone-specific identity number.

FiDe	DoTi	Chlo	LaTe	Site	ClID
1539	18.3	9.0	10.0	EKH	1
807	24.0	9.2	10.0	BJÖ	2
1473	18.7	6.9	10.0	RAM	3
593	28.2	9.4	10.0	ÅKE	4
1493	18.6	17.8	10.0	VÄS	6
1293	19.6	17.0	10.0	LIL	7
1547	18.3	13.5	10.0	KOP	8
1127	20.7	9.0	10.0	EKH	10
820	23.9	9.2	10.0	BJÖ	11
1220	20.1	6.9	10.0	RAM	12
2647	15.4	9.4	10.0	ÅKE	13
740	25.1	10.7	10.0	HÄL	14
1780	17.4	17.0	10.0	LIL	15
1153	20.6	13.5	10.0	KOP	17
1507	18.5	9.2	10.0	BJÖ	20
1240	20.0	6.9	10.0	RAM	21
587	28.4	9.4	10.0	ÅKE	22
707	25.8	10.7	10.0	HÄL	23
3567	14.1	17.8	10.0	VÄS	24
3087	14.7	17.0	10.0	LIL	25
1660	18.0	9.0	10.0	EKH	28
1047	21.5	9.2	10.0	BJÖ	29
5013	12.9	6.9	10.0	RAM	30
613	28.2	9.4	10.0	ÅKE	31
3133	14.6	10.7	10.0	HÄL	32
3273	14.7	17.8	10.0	VÄS	33
1813	17.7	17.0	10.0	LIL	34
1927	17.3	9.0	10.0	EKH	37
3467	14.5	9.2	10.0	BJÖ	38
2773	155	9.4	10.0	ÅKE	40
1387	19.0	10.7	10.0	HÄL	41
3153	14.9	17.8	10.0	VÄS	42
2267	16.5	13.5	10.0	KOP	44
1040	21.4	9.0	12.5	EKH	1
4852	12.7	9.2	12.5	BJÖ	2
1840	17.2	6.9	12.5	RAM	3
1408	19.0	9.4	12.5	ÅKE	4
760	24.0	17.8	12.5	VÄS	6
1480	18.6	17.0	12.5	LIL	7

5780	12.3	13.5	12.5	KOP	8
1820	17.4	9.0	12.5	EKH	10
3070	14.7	9.2	12.5	BJÖ	11
2175	16.4	6.9	12.5	RAM	12
2045	16.7	9.4	12.5	ÅKE	13
2555	14.4	10.7	12.5	HÄL	14
660	26.9	17.0	12.5	LIL	15
1010	21.9	13.5	12.5	KOP	17
865	23.5	9.0	12.5	EKH	19
2955	15.0	9.2	12.5	BJÖ	20
3060	14.4	6.9	12.5	RAM	21
3355	14.5	9.4	12.5	ÅKE	22
4230	13.6	10.7	12.5	HÄL	23
3870	14.1	17.8	12.5	VÄS	24
2165	16.7	17.0	12.5	LIL	25
1870	17.6	9.0	12.5	EKH	28
1104	21.5	9.2	12.5	BJÖ	29
2250	16.6	6.9	12.5	RAM	30
4380	13.7	9.4	12.5	ÅKE	31
1480	19.3	10.7	12.5	HÄL	32
2400	16.3	17.8	12.5	VÄS	33
1350	19.9	17.0	12.5	LIL	34
2080	17.0	9.0	12.5	EKH	37
1720	18.0	9.2	12.5	BJÖ	38
1690	18.1	9.4	12.5	ÅKE	40
2290	16.3	10.7	12.5	HÄL	41
740	25.6	17.8	12.5	VÄS	42
1610	18.5	13.5	12.5	KOP	44
5860	12.3	9.0	15.0	EKH	1
2080	16.5	9.2	15.0	BJÖ	2
6160	12.1	6.9	15.0	RAM	3
1840	17.1	9.4	15.0	ÅKE	4
6500	11.9	17.8	15.0	VÄS	6
5880	12.2	17.0	15.0	LIL	7
5460	12.4	13.5	15.0	KOP	8
4000	13.4	9.0	15.0	EKH	10
2400	15.5	9.2	15.0	BJÖ	11
4740	12.8	6.9	15.0	RAM	12
8960	11.0	9.4	15.0	ÅKE	13
12840	10.3	10.7	15.0	HÄL	14
5280	12.5	17.8	15.0	VÄS	15
6220	12.0	13.5	15.0	KOP	17
2620	15.2	9.0	15.0	EKH	19
5620	12.4	9.2	15.0	BJÖ	20
4140	13.3	6.9	15.0	RAM	21
7200	11.7	9.4	15.0	ÅKE	22

12840	10.3	10.7	15.0	HÄL	23
6640	11.9	17.8	15.0	VÄS	24
1860	17.1	17.0	15.0	LIL	25
9500	11.0	9.0	15.0	EKH	28
1740	17.5	9.2	15.0	BJÖ	29
5360	12.6	6.9	15.0	RAM	30
5580	12.5	9.4	15.0	ÅKE	31
2080	16.5	10.7	15.0	HÄL	32
8300	11.4	17.8	15.0	VÄS	33
8240	11.4	17.0	15.0	LIL	34
2020	16.7	9.0	15.0	EKH	37
3600	14.0	9.2	15.0	BJÖ	38
5900	12.3	9.4	15.0	ÅKE	40
3300	14.3	10.7	15.0	HÄL	41
5920	12.3	17.8	15.0	VÄS	42
2700	15.2	13.5	15.0	KOP	44

Table S3. Field samples 2017. Site, week, temperature (°C), Chlorophyll a (ug per L), and cell density of *S. marinoi* (cells per ml).

Site	Week	TempW	ChlaW	aSKE
VÄS	16	6.2	8.4	2.8×10^{7}
LIL	16	6.3	4.2	3.9×10^{7}
KOP	16	7.0	5.7	1.4×10^{7}
ÅKE	16	7.0	6.4	1.8×10^{7}
HÄL	16	6.9	4.0	3.4×10^{7}
RAM	16	7.3	3.9	0
BJÖ	16	7.1	4.8	1.2×10^{7}
EKH	16	6.8	6.9	4.9×10^{7}
VÄS	18	9.5	3.7	1.4×10^{8}
LIL	18	9.2	2.7	1.6×10^{8}
KOP	18	10.3	5.1	0
ÅKE	18	10.6	9.2	5.4×10^{7}
HÄL	18	10.8	4.4	1.3×10^{7}
RAM	18	10.5	3.9	5.2×10^{7}
BJÖ	18	10.7	1.7	8.2×10^{6}
EKH	18	10.2	2.0	0
VÄS	20	11.6	6.6	7.9×10^{7}
LIL	20	11.6	5.1	4.2×10^{7}
KOP	20	12.9	8.5	0
ÅKE	20	12.1	10.4	1.5×10^{7}
HÄL	20	10.2	10.9	0
RAM	20	12.3	6.0	2.2×10^{7}
BJÖ	20	11.9	3.6	9.9×10^{6}
EKH	20	12.0	18.0	7.0×10^{6}
VÄS	22	16.1	5.6	6.2×10^{6}
LIL	22	16.0	7.3	0
KOP	22	16.8	9.6	0
ÅKE	22	14.3	8.7	0
HÄL	22	13.6	6.6	0
RAM	22	15.8	8.8	6.2×10^{6}
BJÖ	22	15.8	6.1	2.0×10^{7}
EKH	22	14.7	9.8	0
VÄS	24	16.6	7.8	1.7×10^{7}
LIL	24	16.2	7.3	0
KOP	24	17.1	12.3	0
ÅKE	24	16.1	8.4	0
HÄL	24	16.2	7.4	6.6×10^{6}
RAM	24	16.8	5.8	0
BJÖ	24	16.8	6.0	7.3×10^{7}
EKH	24	15.6	9.5	6.6×10^{6}
VÄS	26	16.3	13.8	0

LIL	26	16.0	9.6	6.2×10^{6}
KOP	26	16.5	7.1	0
ÅKE	26	16.4	6.9	0
HÄL	26	16.7	7.2	0
RAM	26	17.7	6.3	9.9×10^{6}
BJÖ	26	17.0	8.4	0
EKH	26	15.5	6.4	0
